# Transcriptome Analysis of Post-Mortem Brain Tissue Reveals Up-Regulation of the Complement Cascade in a Subgroup of Schizophrenia Patients

**DOI:** 10.3390/genes12081242

**Published:** 2021-08-13

**Authors:** Eva Lindholm Carlström, Adnan Niazi, Mitra Etemadikhah, Jonatan Halvardson, Stefan Enroth, Craig A. Stockmeier, Grazyna Rajkowska, Bo Nilsson, Lars Feuk

**Affiliations:** 1 Department of Immunology, Genetics and Pathology, Science for Life Laboratory, Uppsala University, 751 85 Uppsala, Sweden; eva.lindholm@igp.uu.se (E.L.C.); Adnan.Niazi@slu.se (A.N.); Mitra.Etemadikhah@igp.uu.se (M.E.); jonatan.halvardson@igp.uu.se (J.H.); stefan.enroth@igp.uu.se (S.E.); bo.nilsson@igp.uu.se (B.N.); 2 Department of Psychiatry and Human Behavior, University of Mississippi Medical Center, Jackson, MS 39216, USA; cstockmeier@umc.edu (C.A.S.); grajkowska@umc.edu (G.R.)

**Keywords:** complement cascade, schizophrenia, brain tissue, transcriptome

## Abstract

Schizophrenia is a genetically complex neuropsychiatric disorder with largely unresolved mechanisms of pathology. Identification of genes and pathways associated with schizophrenia is important for understanding the development, progression and treatment of schizophrenia. In this study, pathways associated with schizophrenia were explored at the level of gene expression. The study included post-mortem brain tissue samples from 68 schizophrenia patients and 44 age and sex-matched control subjects. Whole transcriptome poly-A selected paired-end RNA sequencing was performed on tissue from the prefrontal cortex and orbitofrontal cortex. RNA expression differences were detected between case and control individuals, focusing both on single genes and pathways. The results were validated with RT-qPCR. Significant differential expression between patient and controls groups was found for 71 genes. Gene ontology analysis of differentially expressed genes revealed an up-regulation of multiple genes in immune response among the patients (corrected *p*-value = 0.004). Several genes in the category belong to the complement system, including *C1R*, *C1S*, *C7*, *FCN3*, *SERPING1*, *C4A* and *CFI*. The increased complement expression is primarily driven by a subgroup of patients with increased expression of immune/inflammatory response genes, pointing to important differences in disease etiology within the patient group. Weighted gene co-expression network analysis highlighted networks associated with both synaptic transmission and activation of the immune response. Our results demonstrate the importance of immune-related pathways in schizophrenia and provide evidence for elevated expression of the complement cascade as an important pathway in schizophrenia pathology.

## 1. Introduction

Schizophrenia (SCZ) affects roughly 0.7% of the adult population. It is a complex neuropsychiatric disorder with both genetic and environmental risk factors. The heritability estimates are as high as 80% [1]. Genetic analyses point to a complex and highly heterogeneous genetic etiology. With the implementation of large-scale genome-wide association studies (GWAS) more than 100 loci were associated with SCZ [2]. Previous genome-wide surveys have also revealed a greater burden of large, rare copy number variations (CNVs) and an increased rate of de novo point mutations among patients [3,4,5,6]. Overall, genetic studies indicate that SCZ is highly polygenic, with risk alleles ranging from common variants with minor risk contribution to rare variants conferring significant risk. The major functional gene categories implicated by GWAS include neuronal, immune and histone pathways [7].

The complex genetic etiology is also reflected in studies of gene expression in SCZ. Early expression analyses in SCZ implicate several pathways, including synaptic function, cell adhesion, apoptosis, as well as the immune system [8,9,10]. The different results may in part be explained by the heterogeneity of SCZ, but also by the small sample sizes of early studies. The first transcriptome study of a larger cohort of SCZ post-mortem brain samples pointed primarily to pathways related to synapse function previously indicated by GWAS results [11]. All significantly differentially expressed genes (DEGs) show a limited fold change, reflecting the heterogeneous and complex nature of the disorder. More recent large cohort gene expression analyses have confirmed previous findings and show that the major expression differences affect genes involved in neural function, but also define specific neural-immune signatures [12].

The strongest GWAS signal in SCZ lies in the major histocompatibility complex (MHC) region, playing a central role in immunity. Subsequent work has shown that a strong genetic risk factor in the MHC region is due to complex genetic variation at the *C4* locus [13]. The *C4* locus, encoding C4A and C4B, is encompassed by a multi-allelic copy number variant (CNV). 

Increased copy number and correlated increased gene expression of C4A are associated with an increased risk for SCZ [13]. *C4A* encodes complement factor 4, which plays an important role in the classical complement pathway, which is one of three key pathways in the innate immune system. The classical complement pathway plays an important role in early microbial defense but is also involved in the removal of cellular debris including a function in synaptic pruning [14,15,16]. A dysregulated pruning process has long been suggested to be important in SCZ pathology [17]. Despite the strong genetic and transcriptional evidence that *C4* plays an important role as a risk factor in SCZ, the exact mechanism of its role in pathogenesis remains unclear.

To further investigate gene expression patterns in SCZ, we performed transcriptome sequencing of snap-frozen post-mortem frontal cortex tissue from 68 SCZ patients and 44 matched control subjects. We performed differential expression analysis to identify genes and processes with altered expression in brain tissue from SCZ patients. Our results provide evidence for up-regulation of the classical complement cascade in post-mortem brain tissue in patients and implicate complement signaling as an important pathway in SCZ pathology. 

## 2. Materials and Methods

### 2.1. Samples

Post-mortem brain tissue samples from 73 patients diagnosed with SCZ and 52 matched control individuals, were obtained from international brain banks (Harvard Brain Tissue Resource Center, MRC Edinburgh Brain Bank, the University of Mississippi Medical Center and the London Brain Bank), with diagnosed individuals and controls matched for age and sex for each brain bank. For the samples from the University of Mississippi Medical Center (UMMC), psychiatric assessments are outlined in Zhu et. al [18]. The majority of the samples (*n* = 107) were from the dorsolateral prefrontal cortex (Brodmann areas 8 and 9) and 18 samples were from the orbitofrontal cortex (Brodmann areas 11 and 12). From these 125 samples, 13 were excluded based on poor RNA quality (RIN < 5) and 112 samples were included in the analysis in the present study. An overview of demographic data for age, sex, brain region, pH, post mortem interval (PMI) and RNA Integrity (RIN) for the patient and the control groups are summarized in Table 1, with details for each individual sample listed in Table S1. The use of the samples in this study was approved by the Regional Ethical Review Board in Uppsala (Dnr 2012/082).

### 2.2. RNA Extraction and Sequencing

Fresh frozen tissue samples were molded in Optimal Cutting Temperature (OCT) and 15 sections (10 µm) were sliced with a cryostat microtome, and immediately placed in QIAzol Lysis Reagent (Qiagen, Hilden, Germany). RNA was extracted using RNeasy Lipid Tissue Mini Kit (Qiagen, Hilden, Germany). DNase treatment was performed with TURBO DNA-freeTM Kit (Life Technologies, Carlsbad, CA, USA). RNA concentration and RIN-values were measured using Agilent 2100 Bioanalyzer. Samples with RIN values > 5 were included in the study (in total 112 patient and control samples). To control for biological and technical variation, extractions were performed on independently sliced duplicates (and a small number of triplicates). In total, 223 libraries were sequenced at the SNP and SEQ Technology Platform at Uppsala University. The RNA preparation and transcriptome sequencing were randomized with regard to replicates, affection status and collection site. The samples were sent for sequencing in eight different batches, as indicated in Table S1. For 13 individuals batch information is missing in Table S1, as those samples did not meet the quality requirements for sequencing (RIN < 5) and were excluded from the analysis. Libraries were prepared using Illumina standard protocols for poly-A selected strand-specific sequencing. Sequencing was performed using Illumina HiSeq 2500 (Illumina, San Diego, CA, USA) which generated 100 bp paired-end reads with a sequencing depth of about 30 million reads per sample. Of 223 libraries from 112 individuals, 95 were sequenced in duplicate, 8 in triplicate and 9 were sequenced only once. All reads were mapped to the hg19 version of the human genome using STAR RNA-seq aligner [19]. Reads from duplicate (and triplicate) libraries were added together for differential expression analysis.

### 2.3. Transcriptome Analysis

For differential expression analysis, reads were counted using the python framework HTseq [20]. High concordance rate was observed among duplicate samples as the mean of correlations between duplicates was R^2^ = 0.93. Next, reads from technical replicates were summed gene wise and genes with <2 mapped reads in 40% of the samples (the size of the control group) were removed. The R library DESeq2 (v 1.28.1) was used for differential expression analysis [21]. The analysis was performed with age, sex and post-mortem interval included as co-variates. After differential expression analysis, the resulting *p*-values were adjusted for multiple testing using the Benjamini–Hochberg procedure. To be able to count reads for the gene *C4A*, which exists in several identical copies in the hg19 reference genome, we also counted multi-mapped reads by filtering on NH:i:2 in the CIGAR string in the BAM file to get multi-mapping reads that only mapped twice.

### 2.4. Cluster Analysis

The counts for each significantly differentially expressed gene for each individual were transformed using the regularized log transform as implemented in the DESeq2 package. Following this, a small number of extreme outlier values were detected (19 out of 7952) and these were adjusted to the same scale as the resulting values. A heatmap of the results was then created and k-means clustering was used to identify three distinctly separated clusters. 

Gene ontology (GO) enrichment analysis was performed using GOseq (v 1.22), controlling for gene length biases [22]. As background for the GO analysis, all genes where DESeq2 performed test for differential expression were used. To investigate the gene expression for the significantly expressed complement genes at the level of individuals, the Z-scores for the expression of each gene were calculated and summarized for each sample and plotted, Z-scores = (x − mean(x))/sd(x).

### 2.5. Validation

cDNA was synthesized using 50 ng RNA using Thermo Scientific Maxima First Strand cDNA synthesis kit for RT-qPCR (#K1671, ThermoFisher Scientific, Waltham, MA, USA) according to the manufacturer’s protocol. Reverse transcriptase minus (RT-) and no template control (NTC) were used as negative control reactions. The validation experiments were performed in two steps. First RT-qPCR was performed for eight differentially expressed genes in seven patient samples and seven control individuals from our sample set, using TaqMan^®^ Gene Expression Assays (ThermoFisher Scientific, Waltham, MA, USA) from Thermo Fisher Scientific. The genes were *C1R* (Hs00357637_m1), *C1RL* (Hs00213057_m1), *C1S* (Hs00156159_m1), *C4* (Hs00246758_m1), *C7* (Hs00940408_m1), *CFB* (Hs00156060_m1), *CFI* (Hs00989715_m1) and *FCN3* (Hs00892390_m1). The house-keeping genes *GAPDH* (Hs02758991_g1), *GUSB* (Hs00939627_m1) and *HPRT1* (Hs02800695_m1) were used as controls. Second, we performed qPCR validation in an additional 14 patients and 10 control individuals using the assays for *C1R* and *C7*, i.e., the two genes in the complement pathway found to be the most significant. The patient and control samples included in the validation experiment were matched according to age and sex. Each RT-qPCR reaction contained 2 μL cDNA, 10 μL Taqman Universal Master Mix II (ThermoFisher Scientific, Waltham, MA, USA), no UNG, 1 μL Taqman Assay (ThermoFisher Scientific, Waltham, MA, USA) (20× concentration) and 8 μL nuclease-free water. The RT-qPCR reaction was performed in Applied Biosystems StepOnePlus Real-Time PCR System using the StepOne Software v2.2.2 according to the manufacturer's protocol. All RT-qPCR was performed in triplicate. Analysis was performed using the ΔΔCt method. Statistical significance of RT-qPCR expression differences between patients and control groups was determined using the nonparametric Mann–Whitney–Wilcoxon test. 

### 2.6. Weighted Gene Co-Expression Network Analysis (WGCNA)

We constructed gene co-expression networks using the WCGNA package [23], starting with the normalized expression data for 23,640 genes. Following this, the DESeq2 implementation of variance stabilizing transform was applied on the expression matrix. Briefly, we constructed gene co-expression networks separately from control individuals and subjects with SCZ. To find the correct power (β) to use when constructing the network, soft thresholding powers were calculated for powers 1 to 20 using a signed network. We used an R^2^ cutoff of 0.8, which corresponded to a selection of β = 7 and β = 4 for the control and SCZ networks, respectively. We filtered out “IF” samples from the analysis to remove any bias. A signed co-expression network was then calculated using blockwiseModules, a wrapper function in WGCNA, with standard settings. Poorly connected genes are clustered in the M0 module (grey color).

### 2.7. Network Analysis of the High Expression Cluster

The co-expression network was constructed from frontal cortex BA 8 and 9 samples from the subset of samples with high complement expression (cluster 1) versus controls (cluster 2 and 3), totaling 32 controls and 22 SCZ individuals using a variance stabilized expression matrix of 23,487 genes. A power of 4 was selected as this power gave the best trade-off between network-free topology and connectivity. A signed co-expression network was then calculated using standard settings. Modules were identified from the network and the eigenvectors of each module were correlated with the covariates age, sex, condition (patient or control). 

### 2.8. Module Preservation Analysis

We quantified the preservation of detected modules between control and SCZ co-expression networks by computing network-based preservation statistics. This required two separate analyses, one with the controls-derived network as the reference and the SCZ-based network as the test, and vice versa. We compared the two networks and prioritized module selections for further analysis based on two main categories of preservation statistics, i.e., density and connectivity. Lower values of preservation statistics, <10 in our case, indicate moderate to weak, and <0 indicates non-preservation of the reference module in the test network. The genes in each module were tested for overlap with each gene set using Fisher's exact test.

### 2.9. Analysis of Overlaps with Previously Identified SCZ Genes

Relevant gene sets from WGCNA were tested for overlap with the following sets of genes: differentially expressed genes (DEGs) in the present study, 693 DEGs in SCZ from the CommonMind Consortium (CMC) [11], and curated genetic associations with SCZ [2,6,24,25,26,27,28]. Briefly, the genetic associations with SCZ were derived from 108 discovered in a common variant GWAS [2], 12 CNVs [24], 756 nonsynonymous de novo mutations and 114 rare variants with loss of function, collated from several studies [6,25,26,27,28], and are available at CMC knowledge portal (http://commonmind.org (accessed on 4 July 2017)).

## 3. Results

**Figure 1:**
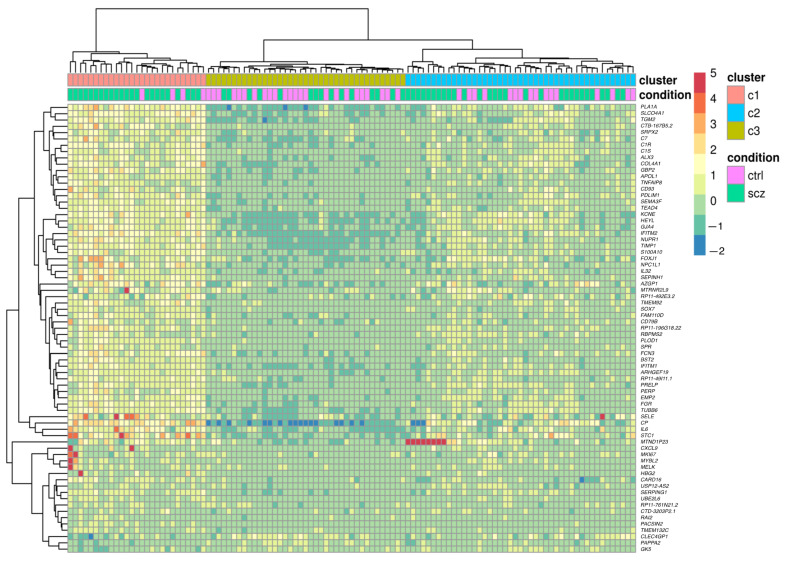
Heat map showing the 71 differentially expressed genes in all samples. Clustering of the genes and samples are indicated at the side and top of the plot, respectively. Patients are denoted in green and controls in pink at the top of the plot. From the plot, three distinct clusters can be seen, with the leftmost cluster containing a majority of immune/inflammatory-related genes.

The sequencing resulted in approximately 20–25 million mapped read pairs per library. Principal component analysis using all expressed genes showed no overall difference between patient and control groups (Figure S1). Differential expression analysis identified 71 significantly differentially expressed genes (DEGs) with an adjusted *p*-value < 0.05 (Table S2). For a more informative classification of genes in terms of function, we performed a Gene Ontology (GO) analysis of the differentially expressed genes. We found immune response (adjusted *p*-value = 0.004) to be the most significantly over-represented category. Cluster analysis of all individuals based on the expression of significant DEGs resulted in three distinct sample clusters (Figure 1). One cluster (Cluster one) showed increased expression of most DEGs and with 23 patients and 4 controls (85% affected), the proportion of affected individuals was significantly higher compared to the other two clusters (*p*-value = 0.002, Fisher's exact test). Comparison of demographics for the individuals between clusters showed that individuals in Cluster one were significantly older compared with subjects in the other two clusters (*p*-value = 0.0008, Mann–Whitney U Test).

### 3.1. Investigation of Genes in the Complement System

**Figure 2:**
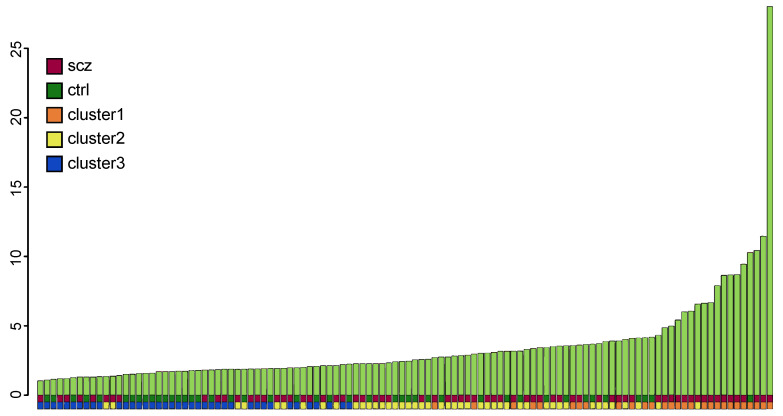
Summarized relative expression of complement genes in patients and controls. The plot is based on the significant differentially expressed complement factors *C1R*, *C1S*, *C7*, *FCN3* and *SERPING1*. The plot shows a clustering of patients among the individuals with the highest complement expression. The bottom row shows which of the three subclusters each sample belongs to. Cluster high (orange) includes most of the samples with high complement gene expression, as compared to cluster median (yellow) and cluster low (blue). The row above shows which samples correspond to affected (brown) and unaffected (green) individuals.

We noted that several of the genes included in the term immune response belong to the complement system. The genes found to be differentially expressed were *C1R*, *C1S*, *C7*, *FCN3* and *SERPING1*. The complement system is one of the most important pathways in the innate immune system and the complement factor C4 has previously been linked to SCZ both at the level of gene copy number and at the level of expression [13]. Since several genes in the complement system were found to show differential expression between patients and controls, this pathway was investigated in more detail. We explored the up-regulation of differentially expressed complement genes at the individual level. Among the 20 individuals with the highest expression, 80% were patients, and they primarily belonged to Cluster one described above (Figure 2).

**Figure 3:**
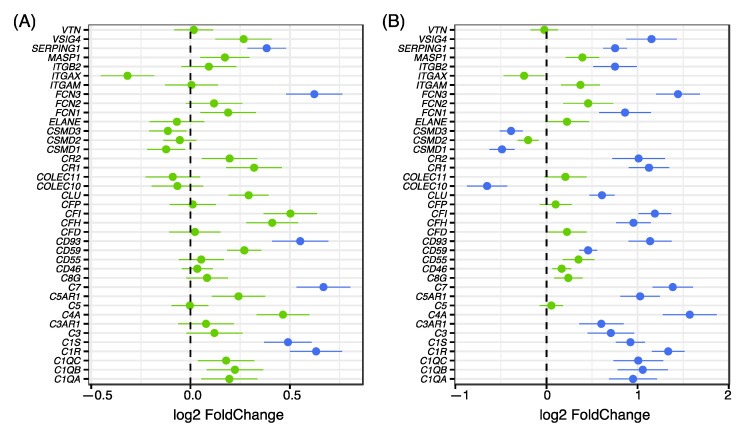
Expression fold changes between cases and controls for all complement genes. (**A**) Genes indicated in blue are significantly differentially expressed (adjusted *p*-value < 0.05) while green color indicates genes that are not differentially expressed. Positive fold changes indicate a higher expression amongst patients. (**B**) Plot showing the fold changes between cases in the high cluster compared to controls for core complement genes. Color scheme is the same as 3A. The results show a significantly increased expression in patients from Cluster one for a majority of the complement genes.

To investigate whether additional complement genes showed a difference in expression despite not reaching statistical significance in the global analysis, the expression of all genes in the complement system included in HUGO Gene Nomenclature Committee database (http://www.genenames.org/cgi-bin/genefamilies/set/492 (accessed on 12 June 2018)) was investigated. Focusing our expression analysis on the 56 genes included in the complement gene family we found 39 to be expressed in our brain tissue samples. Investigation of differential expression using only complement genes, a significant up-regulation was noted in several of the complement system members in patients compared to controls (Figure 3A). Analysis of the same genes for the subset of patients in cluster 1, compared with all controls, showed an even stronger shift in complement expression with significantly increased expression for a majority of the complement factors expressed in the brain tissue samples (Figure 3B). 

### 3.2. Validation of Differentially Expressed Genes

**Figure 4:**
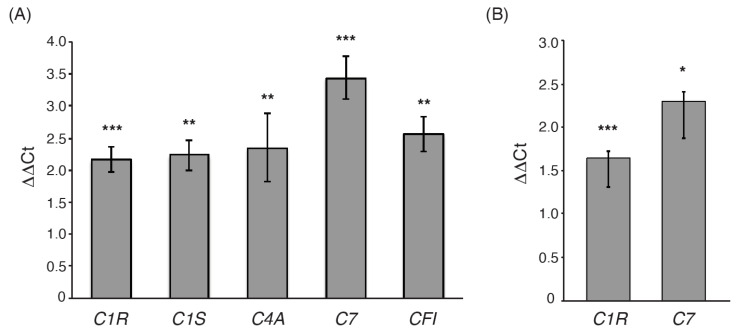
Validation of differential expression of complement genes. (**A**) Quantitative RT-PCR results showing the difference in expression (∆∆CT) for five complement factors *C1R*, *C1S*, *C4A*, *C7*, and *CFI* that were significant or borderline significant in the RNA-seq analysis. Results are based on a comparison of seven patients and seven controls. Error bars show standard error of the mean, and stars indicate level of significance (*p* < 0.01 **, *p* < 0.001 ***). (**B**) Quantitative RT-PCR results showing the difference in expression (∆∆CT) for *C1R* and *C7* in additional 14 patients and 10 controls. Error bars show standard error of the mean, and stars indicate level of significance (*p* < 0.05 *, *p* < 0.01 **, *p* < 0.001 ***).

In order to validate the differential expression of complement factors identified in the RNAseq data, we performed RT-qPCR of five complement genes in seven patients with high complement activation and seven randomly chosen age and sex-matched control samples. All genes showed significantly elevated expression in the patient group (Figure 4A). To increase sample numbers, we performed qPCR in an additional 14 cases with high complement gene expression and 10 age and sex-matched control individuals for *C7* and *C1R*, the two genes with the most significant differential expression in the transcriptome analysis. The results showed significant differences in gene expression levels for both genes, *p*-values 2 × 10^−7^ and 2 × 10^−6^, respectively (Figure 4B), confirming the results from the RNA-seq data. A high correlation between the normalized values from the transcriptome sequencing and RT-qPCR results was observed (R^2^ = 0.85). No signal was detected in the negative controls, neither in the RT- reactions (in which no enzyme was added to the cDNA reaction) nor in the Negative Template Control (in which no template was added to the cDNA reaction).

One potential cause of gene expression differences between samples could be differences in the composition of cell types in the specific section used for RNA extraction. To investigate whether differences in cell composition could explain the observed gene expression differences, we performed an analysis based on cell type-specific marker expression using the tools Cibersort (https://cibersort.stanford.edu/ (accessed on 9 August 2021)) [29] and CellMix (v 1.53) [30]. Both tools showed similar results, indicating that there are no significant differences in cell-type proportions for the major brain cell types (Figure S2) between sample groups.

### 3.3. Network Analysis

To explore coordinated changes in gene expression that may not reach significance at the single-gene level, we performed Weighted Gene Co-expression Network Analysis (WGCNA), first comparing affected individuals with the unaffected controls. None of the modules obtained in the network analysis reached a *p*-value of <0.05. However, we note that the module with the lowest *p*-value (*p* = 0.1) included four (*C7*, *C1R*, *C1S* and *CFI*) of the differentially expressed complement genes.

**Figure 5:**
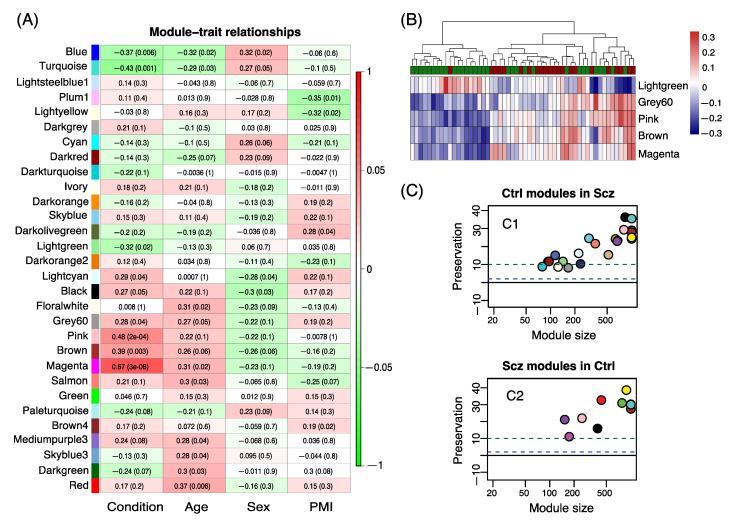
Co-expression gene network analysis and modules preservation. (**A**) Co-expression networks heatmap identified by WGCNA. Each module is assigned a color to the left. All considered traits are mentioned below each column of the heatmap. The figure shows correlation values of co-expression module for condition, age, sex and PMI. *p*-values are listed in parenthesis beside the expression value for each module-trait relationship. (**B**) Eigengene expression for modules from WGCNA analysis of patients in high expression cluster and controls. For each module, eigengene was calculated to represent the overall expression profile of the genes in that module. In the top row, the controls are shown in green and the patients in red. Increased expression is colored in red and decreased expression in blue. Only module light-green, containing many genes related to synapse function, shows decreased expression in patients. (**C**) Dot-plot showing module preservation scores for control modules in SCZ (**C1**) and SCZ modules in controls (**C2**). Lower values of preservation statistics < 10 indicates moderate to weak preservation (indicated by an upper dashed line in each graph) and <0 indicates non-preservation of the reference module in the test network. Of the control networks, 6/25 showed lowered preservation in SCZ, while 1/12 SCZ modules showed low preservation in controls.

We then focused on the subgroup of patients in Cluster one and performed WGCNA analysis on patient samples in Cluster one against control individuals in Cluster two and three. The analysis resulted in five significant modules that were associated with the condition (Figure 5A). The two most significant modules (magenta and pink) showed association with GO terms related to immune response and blood vessel morphogenesis. The magenta module also showed a significant association with age, in addition to affected status (condition). In the significant module “lightgreen”, we observed GO terms related to synaptic transmission, a biological process that was associated with SCZ in multiple previous studies. For each module, the eigengene was calculated to represent the overall expression profile of the genes in a certain module. The expression level of module “lightgreen”, with genes related to synaptic transmission, was decreased in SCZ cases compared to controls, whereas expression levels of other modules were increased in cases (Figure 5B).

### 3.4. Module Preservation Analysis

In order to assess disease-dependent changes in co-expression for modules of interest, we constructed gene co-expression networks separately for control and patient groups. The co-expression network generated from the controls and SCZ individuals consisted of 24 and 11 modules, respectively. We selected modules from control-derived networks that were perturbed in the SCZ group based on their low density or connectivity preservation statistics (Z-score < 10), resulting in six modules (Figure S3). GO analysis of the six modules highlighted genes involved in biological processes including brain/CNS development, synaptic transmission, cell–cell adhesion and blood vessel development. Conversely, only one module could be selected from SCZ-derived networks (Figure 5C and Figure S3) with “Regulation of mTOR” as the most significant GO term.

### 3.5. Gene Set Enrichment Analysis

The genes within each significant module were used in an enrichment analysis to detect whether they were enriched for genes previously associated with or implicated in SCZ, including genes from GWAS studies and rare CNVs or SNVs identified in patients. The genes in the modules were also compared to the differentially expressed genes observed in this study. The results are summarized in Table S3. The most significant module (Module magenta), identified in cluster 1, which included GO terms involved in immune response, was significantly enriched for genes that were differentially expressed in our study (*p*-value 2 × 10^−111^), but not for genes that were genetically associated with SCZ in previous studies. The module pink, which included genes in blood vessel development, was enriched for genes reported to have an increased burden of nonsynonymous variants in SCZ (*p*-value 9 × 10^−4^) and was also enriched for DEGs in our study (*p*-value 5 × 10^−4^).

Gene set enrichment was also performed for the modules found to be less preserved among the patients. Module lightgreen included GO terms related to synaptic transmission, similar to one of the modules obtained in the WGCNA analysis. This module was enriched for genes observed in GWAS studies (*p*-value 4 × 10^−3^). The less preserved modules that included genes with GO terms cell–cell adhesion, blood vessel morphogenesis and brain/CNS development were found to be enriched for genes with rare CNVs and non-synonymous SNVs (Table S4).

## 4. Discussion

The use of transcriptome sequencing to identify differentially expressed genes has proven to be an effective way to identify genes involved in the pathophysiology of complex diseases [31]. Here we used transcriptome sequencing in post mortem brain tissue to identify genes differentially expressed in SCZ patients compared to controls. We report 71 differentially expressed genes, many with a function in the immune system and inflammatory processes. Increased expression of several complement factors was detected in the SCZ group. We show that this signal is driven by a sub-group of patients exhibiting over-expression of the complement cascade.

Clustering based on the differentially expressed genes yielded three sub-clusters, with a very different distribution of cases and controls. We found that this effect is largely driven by the differential expression of several genes involved in the immune response. The fact that immune response is such a strong factor in the clustering of the patients may reflect differences in genetics or exposure to environmental risk. It is noteworthy that a small number of controls also show over-expression of complement factors. Similar subgroups of patients with and without a strong immune response were described in other studies of brain tissue from individuals with SCZ [32,33,34,35]. There are other disorders that are associated with increased complement activation in the brain, including Alzheimer's disease [36,37], Parkinson’s disease [38] and stroke [39]. It is possible that the control individuals showing high complement expression had mild or pre-clinical forms of conditions associated with increased complement activation. The subgroups within the patient populations may either indicate differences in mechanisms of causation or differential response to long-term pathology and treatments. In our data, we cannot readily differentiate between cause and effect.

The complement system is central to the innate immune response and comprises more than 30 proteins. The complement activation can be initiated by three pathways [40], the classical pathway, the mannose-binding lectin pathway and the alternative pathway and is important for eliminating pathogens [41]. Several factors of the complement cascade play a crucial role in synaptic pruning and neuronal plasticity [14,15,16]. It was shown that a higher gene copy number of *C4A* is associated with an increased risk for SCZ and that patients have a higher expression of *C4A* compared to controls [13]. Our RT-qPCR results confirm the increased expression of *C4A* in patients, but we also find increased expression of several other complement factors, especially early factors in the classical pathway. A study by Purves-Tyson et al. also reported increased expression of activators and mediators (*C1Q*, *C3* and *C4*) in the classical pathway in the midbrain of individuals with SCZ [42]. Interestingly, they found this increase to be strongly associated with a subgroup of patients with high inflammatory biotype, i.e., a finding very similar to what we report here. These results highlight the importance of the classical complement cascade in SCZ pathology. 

A recent study aiming to further elucidate the role of the complement system in SCZ show that except for the *C4* locus, there are no other genes in the classical complement pathway that are genetically associated with affected status [43]. However, gene co-expression analysis using *C4A* as a seed gene showed that genes positively co-expressed with *C4A* included genes involved in immune processes and cytokine response. Meanwhile, genes that were negatively co-expressed with *C4A* included synaptic signaling genes, and the authors showed that the genetic risk is driven by the genes that are negatively co-expressed with *C4A*. Thus, similar to our results, an increased expression of complement genes is found especially for early classical pathway genes, with the strongest up-regulation in the frontal cortex. 

There have been several previous studies of gene expression in SCZ brain tissue samples. A comparison of our results with published studies shows that we can replicate many genes previously reported as differentially expressed. The overlap with earlier studies serves as a confirmation of the quality of our data and materials. Of the 71 differentially expressed genes we report, at least ten (Table S2) were found to be significantly dysregulated in SCZ in one or more previous gene expression studies [10,44,45,46,47,48,49,50]. We note that many of the differentially expressed genes related to the immune system and inflammatory response were also reported by Hwang et al., in a study targeting post-mortem hippocampus tissue [10]. However, that study did not report a significant over-expression of genes in the classical complement cascade. Certain members of the complement system have previously been identified as differentially expressed in SCZ patient brain tissue, including the original study reporting increased expression of *C4A* [24], and an earlier study finding increased *FCN3* and *MASP2* expression to be associated with SCZ [9]. 

Many previous gene expression studies using brain tissue from SCZ patients and unaffected individuals suffer from limitations that are also relevant in our study. While brain tissue is highly relevant to study to elucidate SCZ etiology, the RNA will be affected by processes that are initiated post-mortem. While we correct for post-mortem interval and RIN value, the handling of the tissue samples after collection may affect the transcriptome. Since our samples are from international brain tissue banks, we have no insight into the detailed handling of samples during and after collection. There are similar limitations when it comes to details concerning patient selection and inclusion criteria. Potential effects of such confounding factors may be overcome or better corrected for using larger patient and control cohorts, as was shown in some recent large collaborative consortium projects. 

In the CommonMind study by Fromer et al., analyzing transcriptome sequencing of post-mortem tissue from SCZ and control individuals, a strong correlation between the SCZ risk alleles in the *C4* gene and an up-regulation of expression of *C4A* was found [11]. However, apart from *C4*, there is limited overlap between our results and the results of Fromer et al., especially considering that our results show overlap with many previous gene expression studies of SCZ brain tissue. We find better concordance with the recent results from Kim et al. [43] described above, who found a co-expression network centered around *C4A*. Interestingly, there is better concordance with the CommonMind results for the genes present in significant WGCNA modules and the results from the module preservation analysis. For the significant modules, we also noted a significant overlap with genes previously implicated in GWAS, which was also the main finding for differentially expressed genes in the CommonMind Consortium study. This may be an indication that subtle differences in expression of entire networks better reflect the underlying genetic etiology in moderately sized cohorts such as the one used in the present study. 

As our results point to differential expression being strongest in a specific subgroup of the patients, one potential explanation of our findings is that the DEGs implicated by our results reflect a consequence of long-term pathology rather than causative pathways. Differential expression as a result of pathology would explain the overlap of our results with many previous studies of gene expression in SCZ post-mortem brain tissue, as well as the lack of overlap between our DEGs and previous GWAS findings. This conclusion is also supported by the results from Kim et al, where the positive co-expression network around *C4A* includes many complement genes, while it is the negatively co-expressed genes that include neuronal and synaptic functions and enrichment of genetically associated genes. If the expression of immune and inflammatory genes is the result of pathology in a specific subgroup of patients, it could be relevant to search for blood-based biomarkers to identify such patients, as it could have consequences for treatment, including anti-inflammatory therapeutics.

## 5. Conclusions

Our results implicate the importance of immune response and the complement system in the pathology of SCZ. We show that at the level of gene expression, the classical complement pathway is up-regulated in a subgroup of the patients. Investigation of the cause and effect of the increased complement expression in certain patients will be important to further elucidate the etiology of SCZ. Whether directly causative or a result of other underlying risk factors, our results clearly implicate over-expression of the complement cascade as playing a major role in disease progression in SCZ.

## Figures and Tables

**Table 1:** Overview of demographic data for the patient and control groups.

Demographics	Schizophrenia (SCZ)	Control
Age	52.5 ± 16.9	58.6 ± 15.3
Sex	32F:41M	21F:31M
DLPFC:OFC	67:6	40:12
pH ± s.d	6.5 ± 0.2	6.3 ± 0.1
PMI (hours) ± s.d	32 ± 21.9	38 ± 19.9
Average RIN	6.9 ± 1.1	6.7 ± 1.0

## Data Availability

The datasets generated during and/or analyzed during the current study are available from the European Genotyping Archive (EGA) at Study: EGAS00001004199 and Dataset: EGAD00001005948.

## Supplementary Materials

The following are available online at https://www.mdpi.com/article/10.3390/genes12081242/s1, Figure S1: Principal component analysis, Figure S2: Estimation of the fraction of cell types in case and control groups based on gene expression profiles, Figure S3: Module preservation analysis, Table S1: Information for each individual sample used in this study, Table S2: List of genes tested for differential expression and differentially expressed genes (adj. *p*-value < 0.05), Table S3: Gene set analysis of significant modules from WGCNA of Cluster one vs. controls. Table S4: Gene set analysis of modules significant from module preservation analysis.
