# Supplementary material for: Transcriptome Analysis of Post-Mortem Brain Tissue Reveals Up-Regulation of the Complement Cascade in a Subgroup of Schizophrenia Patients
Source: Genes (Basel). 2021 Aug 13;12(8):1242. doi: 10.3390/genes12081242 (PMC8393670; doi:10.3390/genes12081242)

Supplementary Tables

Table S1 (separate file). Information for each individual sample used in this study.

Table S2 (separate file). List of genes tested for differential expression and differentially expressed genes (adj. p-value < 0.05).

Table S3 (separate file). Gene set analysis of significant modules from WGCNA of cluster 1 vs controls.

Table S4 (separate file). Gene set analysis of modules significant from module preservation analysis.

Supplementary Figures

Figure S1. Principal component analysis. The plot shows cases (blue) and controls (red) with no overall specific grouping between cases and controls.

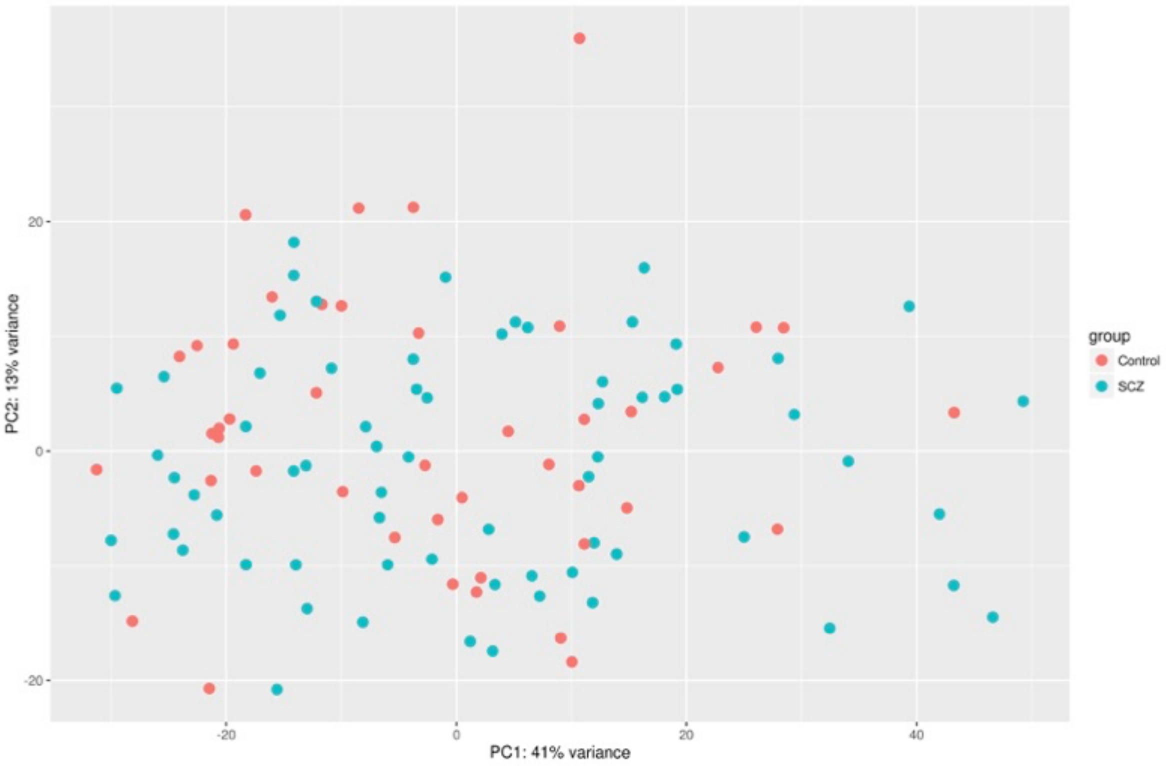

Figure S2. Estimation of the fraction of cell types in case and control groups based on gene expression profiles. Results from two different tools are shown (Cibersort top and CellMix bottom). The results show no significant differences between patients and controls.

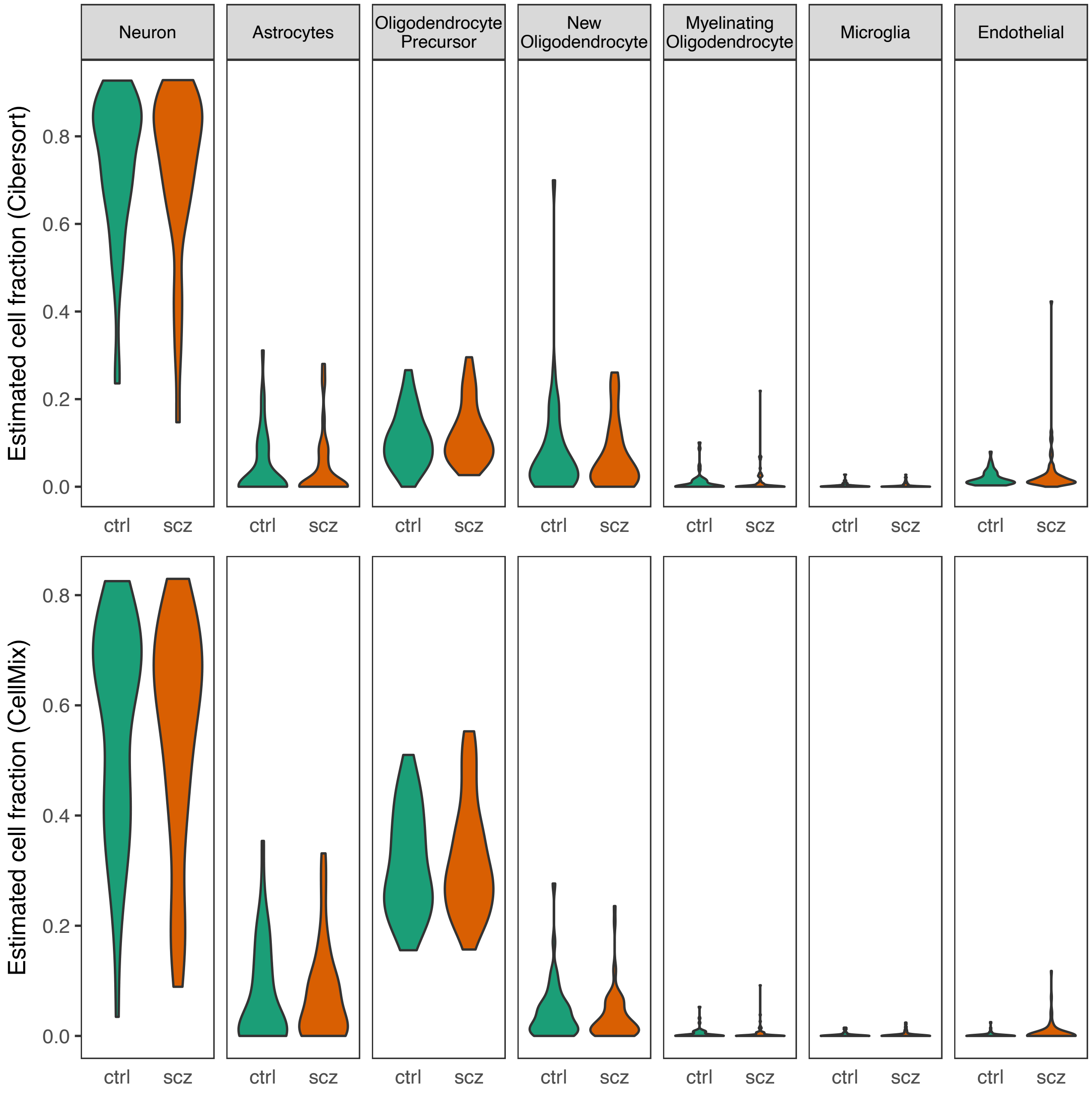

Figure S3. Module preservation analysis. The figure shows module preservation scores predicted from three different approaches for control modules in schizophrenia (top) and schizophrenia modules in controls (bottom).

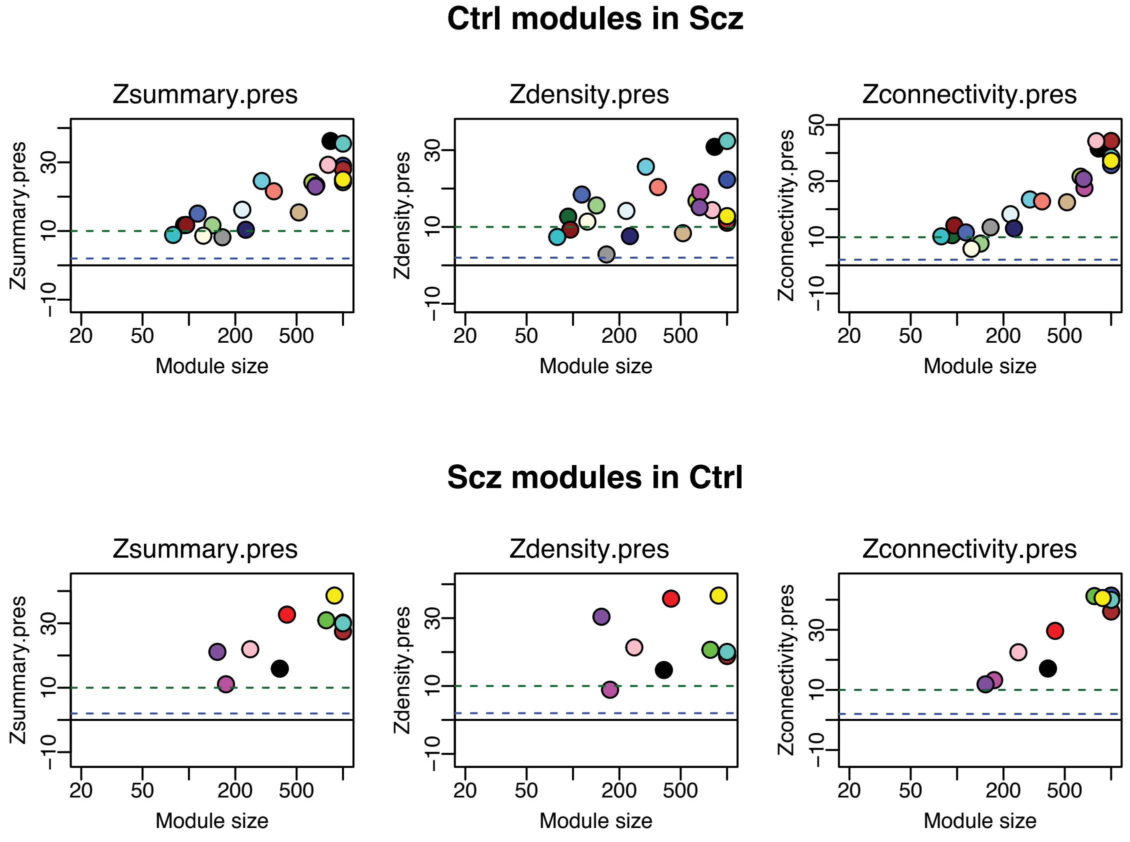

Supplement: Supplementary file 1 [file genes-12-01242-s001.zip › Supplementary Information.pdf]
